# Additional mechanisms conferring genetic susceptibility to Alzheimer’s disease

**DOI:** 10.3389/fncel.2015.00138

**Published:** 2015-04-09

**Authors:** Miguel Calero, Alberto Gómez-Ramos, Olga Calero, Eduardo Soriano, Jesús Avila, Miguel Medina

**Affiliations:** ^1^Centro de Investigación Biomédica en Red sobre Enfermedades NeurodegenerativasMadrid, Spain; ^2^Chronic Disease Programme, Instituto de Salud Carlos IIIMadrid, Spain; ^3^Alzheimer Disease Research Unit, CIEN Foundation, Queen Sofia Foundation Alzheimer CenterMadrid, Spain; ^4^Centro de Biología Molecular Severo Ochoa CSIC-UAMMadrid, Spain; ^5^University of BarcelonaBarcelona, Spain

**Keywords:** Alzheimer, epistasis, exome, GWAS, neurodegeneration, rare variants, risk factors, somatic mutations

## Abstract

Familial Alzheimer’s disease (AD), mostly associated with early onset, is caused by mutations in three genes (*APP*, *PSEN1*, and *PSEN2*) involved in the production of the amyloid β peptide. In contrast, the molecular mechanisms that trigger the most common late onset sporadic AD remain largely unknown. With the implementation of an increasing number of case-control studies and the upcoming of large-scale genome-wide association studies there is a mounting list of genetic risk factors associated with common genetic variants that have been associated with sporadic AD. Besides apolipoprotein E, that presents a strong association with the disease (OR∼4), the rest of these genes have moderate or low degrees of association, with OR ranging from 0.88 to 1.23. Taking together, these genes may account only for a fraction of the attributable AD risk and therefore, rare variants and epistastic gene interactions should be taken into account in order to get the full picture of the genetic risks associated with AD. Here, we review recent whole-exome studies looking for rare variants, somatic brain mutations with a strong association to the disease, and several studies dealing with epistasis as additional mechanisms conferring genetic susceptibility to AD. Altogether, recent evidence underlines the importance of defining molecular and genetic pathways, and networks rather than the contribution of specific genes.

## Introduction

Alzheimer’s disease (AD) is characterized clinically by a gradual decline in memory and other cognitive functions and neuropathologically by gross brain atrophy and accumulation of extracellular amyloid plaques and intracellular neurofibrillary tangles. AD is the most common cause of dementia in the elderly, still without effective treatment. The disease has a strong genetic component and, in a small number of cases, AD segregates as an autosomal dominant trait in families. Although uncommon, the identification of these mutations in the last two or three decades has been very critical not only for diagnosing presymptomatic individuals from autosomal dominant families, but also for important advances in understanding AD pathobiology.

The inheritance of AD exhibits a dichotomous pattern. On one hand, early genetic linkage family studies led to the identification of dominantly inherited, rare mutations in the genes for the amyloid β precursor protein (*APP*)*,* presenilin 1 (*PSEN1*), and presenilin 2 (*PSEN2*) that are associated with early-onset AD (EOAD) with a penetrance close to 100%. Mutations in those three genes account for one third to one half of all autosomal dominant cases, which in turn represent less than 1% of total AD cases (reviewed in Guerreiro et al., 2012). On the other hand, the risk for late onset AD (LOAD), the most common form of the disease (>95 of total cases), is influenced by common variants such as the *APOE* haplotypes, as discussed below (see **Table 1**).

**Table 1:** AD-associated genes.

Gene	Location	Protein	Function
**Rare variants**
*APP*	21q21.3	Amyloid β (A4) precursor protein	• β-amyloid production •Synaptic formation and repair •Neural plasticity
*PSEN1*	14q24.3	Presenilin 1	•β-amyloid production •Notch signaling pathway •Wnt signaling pathway •Endoplasmic reticulum calcium ion homeostasis
*PSEN2*	1q42.13	Presenilin 2	•β-amyloid production •Notch signaling pathway
*ADAM10*	15q22	ADAM metallopeptidase domain 10	•β-amyloid production •Notch signaling pathway
*TREM2*	6p21.1	Triggering receptor expressed on myeloid cells 2	•Inflammatory response
*PLD3*	19q13.2	Phospholipase D family, member 3	•Lipid metabolism
*AKAP9*	7q21-q22	A kinase (PRKA) anchor protein 9	•Cell signaling
GRN*	17q21.32	Granulin	•Signal transduction •Growth factor activity
MAPT*	17q21.1	Microtubule-associated protein tau	•Microtubule cytoskeleton organization
PRNP*	20p13	Prion protein	•Synaptic transmission •Neuronal copper metabolism
**Common variants**
*APOE*	19q13.2	Apolipoprotein E	•Lipid metabolism
*BIN1*	2q14	Bridging integrator 1	•Synaptic vesicle endocitosis •Cytoskeletal dynamics
*CR1*	1q32	Complement component (3b/4b) receptor 1	•Regulation of complement activation
*ABCA7*	19p13.3	ATP-binding cassette, sub-family A (ABC1), member 7	•Lipid metabolism •Phagocytosis
*FERMT2*	14q22.1	Fermitin family member 2	•Cell-cell adhesion •Cell spreading •Cell shape modulation
*HLA-DRB5-DRB1*	6p21.3	Major histocompatibility complex, class II, DR β 5, β 1	•Immune response
*CD2AP*	6p12	CD2-associated protein	•Regulation of actin cytoskeleton reorganization
*PTK2B*	8p21.1	Protein tyrosine kinase 2 β	•Calcium-induced regulation of ion channels •MAP kinase signaling pathway
*INPP5D*	2q37.1	Inositol polyphosphate-5-phosphatase, 145 kDa	•Immune response
*CUGBP-CELF1*	11p11	CUGBP, Elav-like family member 1	•Regulation of RNA splicing
*CD33*	19q13.3	CD33 molecule	•Cell signaling
*SLC24A4-RIN3*	14q32.12	Solute carrier family 24 (sodium/potassium/calcium exchanger), member 4- Ras and Rab interactor 3	•Ion transmembrane transport •Cell signaling •Endocytosis
*ZCWPW1*	7q22.1	Zinc finger, CW type with PWWP domain 1	•Zinc ion binding
*MEF2C*	5q14.3	Myocyte enhancer factor 2C	•Myogenesis
*NME8*	7p14.1	NME/NM23 family member 8	•Ciliary function
*MS4A* cluster	11q12.2	Membrane-spanning 4-domains, subfamily A	•Signal transduction
*EPHA1*	7q34	Ephrin type-A receptor 1	•Immune response •Neural development
*PICALM*	11q14	Phosphatidylinositol binding clathrin assembly protein	•Clathrin-mediated endocytosis
*CLU*	8p21-p12	Clusterin	•Lipid metabolism •Immune response
*CASS4*	20q13.31	Cas scaffolding protein family member 4	•Cell–cell adhesion •Cell spreading
*SORL1*	11q23.2-q24.2	Sortilin-related receptor, L(DLR class) A repeats containing	•Endocytosis •Sorting
*DSG2*	18q12.1	Desmoglein 2	•Cell–cell adhesion

*GRN, MAPT, and PRNP mutations have been found in some individuals with clinical phenotypes indistinguishable from AD (Lee et al., 2014). Variants of genes associated with Alzheimer’s disease (AD) can be classified according to their frequency as rare or common variants. Rare variants are commonly associated with early onset AD, while common variants are associated with late onset Alzheimer’s disease (LOAD).

Over 30 dominant mutations in the *APP* gene (located at chromosome 21q21) account for about 15% of early-onset autosomal dominant cases of AD. Interestingly, two recessive APP mutations, A673V, and E693D, also reportedly cause EOAD (Tomiyama et al., 2008; Di Fede et al., 2009). *APP* encodes a ubiquitously expressed transmembrane protein and most mutations cluster around or within the Aβ domain. Duplications of *APP* and neighboring sequences are also linked to EOAD. Families carrying these duplications exhibit classic AD and cerebral amyloid angiopathy (reviewed in Guerreiro et al., 2012). These copy number mutations display different frequencies depending on the geographic population studied, being much more frequent in Japanese than in Europeans early-onset familial cases (Raux et al., 2005; Sleegers et al., 2006; Blom et al., 2008; Kasuga et al., 2009). Furthermore, patients with Down syndrome, which results from a chromosome 21 trisomy, develop AD neuropathology (Hartley et al., 2014).

Structurally similar integral membrane proteins *PSEN1* and *PSEN2* are part of the γ-secretase protein complex responsible for APP cleavage and Aβ generation (Medina and Dotti, 2003). About 80% of EOAD cases have been reported to carry dominant, pathogenic mutations in *PSEN1* (located at chromosome 14q24.3), whereas approximately 5% have been identified in *PSEN2*, which is located at chromosome 1q31–q42 (Bird, 1999) Mutations in *PSEN1* and *PSEN2* appears scattered throughout the protein, although some clustering can be observed in the transmembrane domains (Guerreiro et al., 2012).

On the other hand, in non-familial, sporadic cases of AD mostly associated with late onset (LOAD), the ε4 allele of the apolipoprotein E (*APOE*) gene has been identified as a major genetic risk factor contributing to the pathogenesis of AD (Corder et al., 1993; Strittmatter et al., 1993). The *APOE* gene is located at chromosome 19q13.2 and encodes a highly pleiotropic glycoprotein (Siest et al., 1995) involved in the transport of cholesterol and other lipids in the periphery and brain (Mahley, 1988). There are three polymorphic alleles (ε2, ε3, and ε4) encoding three isoforms that differ on two amino acid residues (112 and 158): ApoE2, ApoE3 (the most common form), and ApoE4. Presence of the ApoE4 allele increases risk in familial and sporadic EOAD and LOAD, increasing risk threefold when in heterozygosis and up to 15-fold when in homozygosis (Ashford, 2004). The ε4 allele has also a dose-dependent effect on the age at onset. Conversely, the allele ε2 appears to lowers the risk for LOAD and delays age at onset (Corder et al., 1994).

Overall, these four genes account for 30–50% of the inheritability of AD (Jonsson et al., 2012). The advent of genome-wide association studies (GWAS) in recent years has allowed the identification of novel genetic associations. Hence, over 20 genetic loci have been reported to associate with increased susceptibility for LOAD, essentially common variants with a small individual effect on risk (unlike *APOE*), but also recently identified rare susceptibility variants in LOAD with larger effects (see below).

Thus, besides *APOE*, successive GWAS analyses have generated replicable associations with LOAD for several genes: *CLU*, *PICALM*, *CR1*, *BIN1*, *CD33*, *MS4A* cluster, *CD2AP*, *EPHA1*, and *ABCA7* (reviewed in Karch et al., 2014). A recent meta-analysis of GWAS data from four large consortia confirmed these previous associations (except for *CD33*) and reported 12 new susceptibility loci for AD: *CASS4*, *CUGBP-CELF1*, *DSG2*, *FERMT2*, *HLA-DRB5-DRB1*, *INPP5D*, *MEF2C*, *NME8*, *PTK2B*, *SLC24A4-RIN3*, *SORL1*, *ZCWPW1* (Lambert et al., 2013; **Table 1**). These genes increase AD risk in a non-Mendelian fashion, but first-degree relatives of LOAD patients have twice the expected risk of AD and LOAD is more frequent in monozygotic than in dizygotic co-twins (Reitz and Mayeux, 2014). However, the observed risk or protective effects of all the single nucleotide polymorphisms (SNPs) tagging these 21 loci are rather small, with odds ratio (OR) ranging from 1.22 to 0.77 (Karch et al., 2014), and the genetic effect attributable to each of these associated loci had population-attributable fractions or preventive fractions between 1.0 and 8.0% (Lambert et al., 2013). Although these data are of great value to delineate the fundamental physiopathological avenues of the disease, they only explain a small proportion of familial clustering (Manolio et al., 2009). Therefore, strong additional efforts in sequencing and post-GWAS analyses have to be put forward to find the remaining missing heritability in order to completely undercover the genetics of AD, utterly aiming at enabling effective prevention, prediction and treatment of the disease (Manolio et al., 2009; Lambert et al., 2013).

A number of recent excellent reviews have focused on the analysis of common genetic risk factors for LOAD and their role in pathogenesis (Guerreiro et al., 2013a; Lambert et al., 2013; Karch et al., 2014; Reitz and Mayeux, 2014). The genes identified can be classified in a few pathways, mainly lipid metabolism, immune response, endocytosis (Karch and Goate, 2015). Despite the identification of all the above-mentioned loci associated with AD, a large proportion of the genetic component of the disorder remains unexplained (Lord et al., 2014). Using alternative AD phenotypes may serve as a tool to unveil additional genes that could modify particular aspects of the disease (Karch and Goate, 2015). In here, we review additional mechanisms that may at least partially explain this missing heritability such as epistasis, rare variants, or the presence of somatic mutations. In this sense, this review does not intend to comprehend an exhaustive analysis of the literature on these subjects, but to put forward important concepts and the general evolution of the field. The analysis of epigenetic modifications or microRNA studies are out of the scope of the present review.

## Rare Variants

After a decade of intense efforts on GWAS analysis, lately followed by meta-analysis of studies performed on very large cohorts, a number of common variants have been identified to have replicable, although small effects on LOAD with no clear functionality in some cases (Cruchaga et al., 2014); and it is unlikely that many common variants with moderate-large effects remain to be identified (Ridge et al., 2013). However, new rare functional variants, with larger effects are expected to be associated with AD. Thus, researchers from the AD Genetics Consortium argue that more than 25% of phenotypic variance remains unexplained by known markers, but it is tagged by common SNPs, hence suggesting that novel AD markers that account for large amounts of phenotypic variance are likely to be rare (Ridge et al., 2013). Thus, both rare and common variants contribute to AD risk (Guerreiro et al., 2013a; see **Table 1**). However, GWAS are not well suited for the discovery of these rare variants, and the use of new techniques such as exome analysis or whole genome sequencing will be required. Genome and exome sequencing studies in large data sets are likely to add new genes (with a moderate or low association). However, it remains to be seen whether additional pathways can be identified (Karch and Goate, 2015).

Besides the well-characterized mutations on *PSEN1*, *PSEN2,* and *APP* found in pedigrees of familiar EOAD, the advent of new powerful tools for genome analysis is already delivering a more comprehensive identification of rare variants potentially associated with the disease. For example, whole-genome sequence analysis from 1,795 Icelanders has led to the identification for the first time of a coding mutation (A673T) in *APP* that protects against AD and cognitive decline (Jonsson et al., 2012). In a different study, exome sequencing analysis has identified two novel pathogenic *PSEN1* mutations (p.L166V and p.S230R) in British EOAD (Sassi et al., 2014a), although a similar study did not identify novel variants in AD in an Asian population (Chung et al., 2014).

Additionally, rare variants in other genes previously not associated with familiar EOAD have been found to have a high to moderate effect size. Thus, two rare, highly penetrant mutations in *ADAM10* (p.Q170H and p.R181G) for LOAD have been reported (Kim et al., 2009), emphasizing the importance of whole genome or whole exome sequencing approaches to find rare variants causing LOAD, in addition to common variants (Jonsson et al., 2012).

Following whole-exome sequencing and whole-genome sequencing strategies, a rare variant in the *TREM2* gene (p.R47H) has also been associated with an increased risk of AD with an OR of 3.4 (Guerreiro et al., 2013b; Guerreiro and Hardy, 2013; Jonsson et al., 2013). Recently, two more *TREM2* variants have been associated with either increased (p.R62H) or decreased (p.S144G ) risk in AD (Benitez et al., 2014; Cuyvers et al., 2014). Interestingly, *TREM2* also appears to be associated with Parkinson’s disease, frontotemporal dementia (Rayaprolu et al., 2013; Le Ber et al., 2014), and amyotrophic lateral sclerosis (Guerreiro and Hardy, 2013), although this remains controversial (Slattery et al., 2014).

Applying whole exome sequencing with a family-based design aimed at detecting novel AD risk genes, several rare, missense, and synonymous variants in phospholipase D3 (*PLD3*) have been reported to be associated with AD risk (Cruchaga et al., 2014). One variant in *PLD3* (p.V232M), which segregated with some LOAD families appears to increase two- to threefold the risk for AD, perhaps through its influence on APP processing (Cruchaga et al., 2014).

Whole exome sequencing analysis performed on seven African American AD cases, (Logue et al., 2014) has led to find two new rare variants in *AKAP9 (*A-kinase anchor protein nine gene) potentially associated with AD, that in the replica population from the AD Genetics Consortium showed a strong association (rs144662445, OR = 2.75; rs149979685, OR = 3.61).

Recently, the Ibero-American Alzheimer Disease Genetics Group Researchers analyzed the coding region and flanking sequences of *APP*, *PSEN1*, *PSEN2*, *MAPT,* and *GRN* by pooled-DNA exon sequencing in 167 clinical and five autopsy-confirmed, mainly EOAD cases (Jin et al., 2012). Interestingly, pathogenic mutations in *PSEN1*, *GRN,* and *MAPT* genes were found in 2.3% of the screened cases, suggesting that pathogenic mutations or risk variants in MAPT and in GRN are as frequent in clinical AD cases as mutations in *APP*, *PSEN1,* and *PSEN2* (Jin et al., 2012). These findings underscore the pleotropic role of genes such as *MAPT* and *GRN* that can influence both frontotemporal dementia and AD (Jin et al., 2012; Lee et al., 2014; **Table 1**). Likewise, missense or nonsense haplotypes in *PRNP* (p.I215V or p.Q160) further highlight how very similar genotypes in *PRNP* result in strikingly different clinical phenotypes such as prion diseases and AD (Muñoz-Nieto et al., 2013; Guerreiro et al., 2014).

In contrast, the role of rare coding variability in Mendelian inherited dementia genes (*APP*, *PSEN1*, *PSEN2*, *GRN*, *MAPT*, and *PRNP*) has also been investigated in LOAD. Results indicated that rare coding variability in *PSEN1* and *PSEN2* may influence the susceptibility for LOAD, while *GRN*, *MAPT*, and *PRNP* do not appear to be major contributors to LOAD (Sassi et al., 2014b).

## Somatic Mutations

Advanced age is the single most important risk factor for AD. Randomly acquired DNA damage in the nuclear genome is also associated with aging, and post-mitotic neuronal tissue is at special risk of DNA damage due to the elevated production of DNA-damaging reactive oxygen species (ROS) associated with their high metabolism (Barja, 2004; Kennedy et al., 2012). Moreover, the mutation rate of somatic cells is roughly an order of magnitude higher than mutation rates for germ-line cells (Lynch, 2010). On the other hand, evolution cannot put selective pressure on those deleterious mutations that produce defects long after the age of reproduction as it is the case of AD (Medawar, 1952; Kennedy et al., 2012). According to the so called somatic mutation theory of aging, the accumulation of mutations in the genetic material of somatic cells as a function of time results in a decrease in cellular function. A significant amount of research has shown that somatic mutations play an important role in aging and a number of age-related pathologies (Jeppesen et al., 2011; Kennedy et al., 2012). Additionally, some genetic traits may present antagonistic pleiotropy as in the case of *APOE* ε4 variant that appears to be associated with improved perinatal health and survival, favoring the selection of the ε4 allele despite its later life deleterious effects (reviewed in Eisenberg et al., 2010). With this perspective, it is not surprising that somatic cells, especially in the brain, may accumulate a much higher proportion of mutations in certain DNA hot spots (e.g., CpG dinucleotides) that correlate (at a lower scale) with the reported occurrence of pathogenic mutations in germinal-cell lines. The effect of these somatic mutations will depend on stochastic processes that allow the generation of cellular damage that may also propagate to other cells (Aguzzi and Rajendran, 2009). Therefore, in a wide sense, somatic mutations could be considered as rare variants locally generated.

Recent technological advances in the field of genomics and the emergence of next generation sequencing (NGS) techniques have aggravated the difficulties of interpreting GWAS to reveal the genetic basis of brain disorders. Despite a wealth of data on candidate genes affecting the susceptibility to AD and other neurological disorders, their inherent contribution to the pathogenesis and their relationship with non-genetic environmental risk factors is not yet fully understood. Evidence has accumulated showing that somatic variations can affect neuronal populations and may play a role in brain pathogenic processes. Thus, it has been suggested that, at least in some cases copy number variations (CNV) or SNP linked to major AD and other neurological disorders may lead to genetic dysregulation resulting in somatic mosaicism (Iourov et al., 2013). Moreover, environmental factors may affect cellular repair mechanisms and trigger a series of events that end up generating genomic instability.

Increasing evidence has also shown that, even at low levels of mosaicism, somatic mutations can cause neuropsychiatric, and pediatric disorders such as epilepsy, autism, lissencephaly, and intellectual disability. Interestingly, at least some of these cases appears to have been caused by somatic mutations limited to the brain (Poduri et al., 2013). In addition, a number of reports have shown that a significant population of neurons exhibit aneuploidy in both mice and humans and the level of aneuploidy could be as high as 1–3% in adult brain (Rehen et al., 2005). Furthermore, brain tissue from both AD and other disorders exhibits increased levels of aneuploidy when compared to unaffected brain (Iourov et al., 2009; Frade and López-Sánchez, 2010).

Furthermore, a recent study has investigated the presence of tissue-specific exonic single nucleotide variations (SNV), taking blood exome as a control (Gómez-Ramos et al., 2014). Interestingly, the number of SNV per chromosome was independent of chromosome size, but it was suggested to mainly relate to the number of protein-coding genes per chromosome. Although similar patterns of chromosomal distribution of tissue-specific SNVs were found, clear differences were also detected, supporting the notion that each individual tissue has a specific SNV exome signature.

Some neurodegenerative disorders have been associated with or can be affected by somatic mutations. Thus, EOAD has been attributed to a somatic mosaic *PSEN1* mutation present in the brain (Beck et al., 2004). Similarly, a case of sporadic Creutzfeldt–Jakob disease has been reported to be caused by an early embryonic somatic mutation in the *PRNP* gene (Alzualde et al., 2010). Also, patients of disorders caused by the expansion of highly mutable repeats such as Friedrich’s ataxia or Huntington’s disease, can exhibit notable somatic heterogeneity in repeat lengths across different brain regions and tissues (Kahlem and Djian, 2000; Hellenbroich et al., 2001; Møllersen et al., 2010). Age-related somatic mutations, known to play a role in cancer (Jacobs et al., 2012), have also been proposed to be involved in normal aging processes as well as in neurodegeneration (Kennedy et al., 2012). Interestingly, very recent whole-exome sequencing analyses of various tissues from sporadic AD patients have found a remarkably high number of brain-specific SNV in AD hippocampal samples when compared with blood (Parcerisas et al., 2014), suggesting that somatic genetic mosaicism and brain-specific genome reshaping may contribute to the pathogenesis of sporadic AD.

As NGS techniques become more efficient and affordable in the coming years, somatic mosaic mutations could be more readily detected, and somatic mutation rates could be systematically determined across different regions, cell types, and time points of the human brain development. Although the limitation of brain tissue will still limit studies on brain-specific somatic mutations, novel techniques, and improved bioinformatic analyses will allow us to address the role of somatic mosaicism in neurological conditions. Determining whether brain somatic mutation are at least partially responsible for AD and other neurodegenerative disorders will be one of the next major challenges in the field.

## Epistasis

The functional expression of certain genes may be determined by the interaction with other genes, as well as with environment factors. Therefore, some of the missing heritability may be conditioned by epistasis, a crucial factor to understand and interpret genetic pathways and their functional role in complex systems. Epistasis may refer to different phenomena, including the functional interaction between genes or the statistical deviation from additive gene action, among others (Phillips, 2008), yet in the context of this review, we will use the term epistasis as synonym of functional gene–gene interaction. However, GWAS, whole genome and exome sequencing analyses have been mainly focused on standard single-locus tests with different level of precision. With the increasing availability of genetic data delivered by the new genomic technologies, it is becoming clearer the need to address the problems from a holistic position, taking into account the interaction among different players at different levels.

The analysis of epistasis, even if limited to the interaction between two genes, is technically demanding and methodologically limited at present. Part of the challenge for epistasis analysis in GWAS is the magnitude of the search and the computational complexity associated with it (Ritchie, 2015). The methods and related software packages used to detect the interactions between genetic loci that contribute to human genetic disease have been recently re-examined (Cordell, 2009). Lately, several studies have reviewed novel advances in the methodology for detecting epistasis (Ma et al., 2013; Wei et al., 2014) and discussed its relevance in the context of GWAS (Wei et al., 2014). A protocol for exhaustive genome-wide association interaction analysis and applied to AD has been proposed, finding replicable epistasis between the *KHDRBS2* and *CRYL1* gene loci (Gusareva et al., 2014).

A powerful tool to disentangle the complexity of a disorder such as AD is the use of the concept of endophenotypes to define genetic association elements with more elementary phenomena rather than with the whole spectrum of complex diagnostic entities. Therefore, markers of disease (at any level) that correlate with a genetic trait may be useful to explore the pathophysiological mechanisms related to specific aspects of the syndrome (Cannon and Keller, 2006).

Synergy factor (SF) analysis has been used to assess over 100 claims of epistasis in sporadic LOAD, finding 27 gene–gene interactions that were significantly associated with AD (Combarros et al., 2009). Additionally, the authors demonstrated by meta-analysis the interaction of *APOE* ε4 with specific variation in four different genes, namely *ACT*, *BACE1*, *IL6*, and *BCHE* (Combarros et al., 2009). More recently, a systematic review of genetic studies published between 2009 and 2012 by the Genetics Core of the AD Neuroimaging Initiative (ADNI) focused on genetic associations with disease status or quantitative disease endophenotypes including structural and functional neuroimaging, fluid biomarker assays, and cognitive performance. In this review, the authors summarize the association of several AD risk genes with imaging, fluid and cognitive phenotypes, and indicated the association with multiple ADNI phenotypes of several other genes (i.e., *APOC1*, *FTO*, *GRIN2B*, *MAGI2*, and *TOMM40*). The authors suggested the combination of genetic data and phenotypes for targeting future studies employing NGS and convergent multi-omics approaches, and for clinical drug and biomarker development (Shen et al., 2014). Likewise, the use of neuroimaging measures to define powerful quantitative endophenotypes for exploring epistatic relationships in order to explain some of the missing heritability in AD has been proposed (Hohman et al., 2013). Moreover, the analysis of epistasis has also been shown to be a key factor for genomic prediction and its application in preventive strategies through the identification of population strata at increased risk of disease and clinical decision making (Wray et al., 2013).

Many studies are now focused on finding how genetic variants influence specific traits of AD pathology, such as amyloid burden (Kauwe et al., 2011, 2014; Bali et al., 2012; Hohman et al., 2013; Shen et al., 2014), neurofibrilar pathology (Kauwe et al., 2011; Shen et al., 2014), brain atrophy (Shen et al., 2014), cognitive decline (Pedraza et al., 2014), or inflammation (Kauwe et al., 2014). However, the complexity and heterogeneity of AD requires further analysis going beyond the study of single markers in isolation by taking into account interactions between genes. For example, many of the LOAD risk genes do not show single marker associations with amyloid pathology, but only through interaction with other genes (e.g., *BIN1* ×*PICALM*; Hohman et al., 2013).

Therefore, it is important to note that epistasis may be a barrier to uncover the genetic basis of complex disorders, since the effects of quantitative trait loci can be masked by interactions with other loci (Phillips, 2008). As demonstrated for *GSTM3* gene and the *HHEX*/*IDE*/*KIF11* locus, the association with AD could be purely epistatic with neither polymorphism showing an independent effect (Bullock et al., 2013). The same group, involved in the Epistasis Project, has also reported an interaction between transferrin and *HFE* genes, confirming previous findings (Robson et al., 2004) and suggesting that iron overload may be involved in the development of AD (Lehmann et al., 2012).

Arguably, *APOE* is the main genetic risk factor in AD and it is likely to interact with other genes. The epistatic effect for *APOE* has been recognized both in familial EOAD and LOAD modifying the age at onset (Sorbi et al., 1995; Combarros et al., 2009; Hohman et al., 2013). Using whole-trascriptome analysis of brain gene expression, It has been demonstrated that *APOE* ε*4* carrier status was associated with a consistent transcriptomic shift that resembled the LOAD profile (Rhinn et al., 2013). However, unlike the genes underlying familial AD, LOAD susceptibility genes do not specifically alter the Aβ42/40 ratios, suggesting that these genes probably contribute to AD through distinct mechanisms (Bali et al., 2012). Interestingly, a recent study (Naj et al., 2014) has also demonstrated an association of *APOE* variants with age at onset among affected individuals with LOAD and observed novel associations of *CR1*, *BIN1*, and *PICALM* with age at onset.

Along the same lines, we have explored epistasis and pleiotropic effects of various genes and neurodegenerative disorders. AD and Creutzfeldt-Jakob disease (CJD) are now considered both as part of a wider group generically named as conformational disorders, and more specifically brain amyloidosis. Under the assumption that these two disorders share common pathophysiologic mechanisms involving protein aggregation in the brain leading to fatal degeneration, we investigated a possible genetic interaction between *APOE* ε4 allele and the polymorphic codon 129 of the *PRNP* gene in both AD and CJD populations compared to a common control population (Calero et al., 2011). Interestingly, we found a synergistic age-dependent interaction between the two genes (*APOE* × *PRNP*) in both disorders (SF = 3.59, *p* = 0.027 for AD; and SF = 7.26, *p* = 0.005 for CJD). Our data suggest the involvement of common pathways involved in the generation, clearance, and neurotoxic signal transduction of Aβ peptides and PrP in AD and CJD. The finding of an age-dependent interaction underscores the importance of genetic risk analysis stratified according to other potential interacting/confounding factors such age, sex, or comorbidities.

Finally, the comprehensive analysis of high order interactions is limited by the exponential number of potential interactions and our technical capacity; therefore, we need to focus on particular subsets of the interaction space by using additional functional information (Phillips, 2008). However, the analysis of epistasis should not be limited only to candidate genes.

## Conclusion

Over 20 loci have been associated with LOAD, defining three main routes altered in AD: lipid metabolism, immune response, and endocytosis (see **Table 1**). Altogether, these association analyses to different genes by GWAS underscore the importance of defining pathways and networks rather the contribution of specific genes. Future research should be focused on defining shared and specific mechanisms among distinct neurodegenerative and other chronic disorders including cardiovascular disease, metabolic disorders, or even cancer. Further insights into complex disorders such as AD are expected from the integration of different -omics with detailed high-quality clinico-physiological characterization of cases and controls allowing the development of new disease biomarkers and therapeutic avenues, and will enable the implementation of personalized medicine (Ramanan and Saykin, 2013). The identification by GWAS of multiple disease-associated loci of small effect size emphasizes the polygenic nature of the heritability of complex traits and common disorders such as AD, giving rise to approach the disorder as of quantitative dimensions instead of as a qualitative disorder (Plomin et al., 2009).

However, a large proportion of genetic component of the disorder remains unexplained (Lord et al., 2014). As reviewed above, epistasis, rare variants, and the presence of somatic mutations are additional genetic mechanism that may explain in part this missing heritability. Future research should emphasize: (i) the complete characterization of the genetic risk factors including common-low effect size and rare-high effect size variants, (ii) the use of endophenotypes to associate specific traits of the disease to genetic factors and use this information for risk prediction and definition of treatment response groups, (iii) the analysis of epistasis among different risk genes and the interaction with other factors such as age and sex, and (iv) the potential pleiotropism associated with certain loci that may confer either risk or protection to AD and may transversally regulate different neurodegenerative disorders. From a practical point of view, it is important to acknowledge that most AD patients usually present a combined pathology with other chronic disorders including cardiovascular disease or metabolic disorders such as diabetes mellitus type 2; and therefore we should investigate how these pathologies interact with each other, in order to properly treat each individual patient.

## Conflict of Interest Statement

The authors declare that the research was conducted in the absence of any commercial or financial relationships that could be construed as a potential conflict of interest.
